# The quality of systematic reviews/meta‐analyses assessing the effects of ankle–foot orthosis on clinical outcomes in stroke patients: A methodological systematic review

**DOI:** 10.1002/hsr2.1130

**Published:** 2023-03-01

**Authors:** Saeed Shahabi, Parviz Mojgani, Kamran Bagheri Lankarani, Maryam Jalali

**Affiliations:** ^1^ Health Policy Research Center, Institute of Health Shiraz University of Medical Sciences Shiraz Iran; ^2^ Iran‐Helal Institute of Applied Science and Technology Tehran Iran; ^3^ Research Center for Emergency and Disaster Resilience Red Crescent Society of The Islamic Republic of Iran Tehran Iran; ^4^ Rehabilitation Research Center, Department of Orthotics and Prosthetics, School of Rehabilitation Sciences Iran University of Medical Sciences Tehran Iran

**Keywords:** evidence‐based practice, methodological quality, orthotic devices, rehabilitation, stroke, systematic review

## Abstract

**Background and Aims:**

Given the importance of systematic reviews (SRs) for practitioners, researchers, and policymakers, it is essential to assess them to ensure robust methodology and reliable results before applying them. The purpose of this methodological study was to assess the methodological and reporting quality of recently published SRs and/or meta‐analyses (MAs) evaluating the effects of ankle–foot orthoses (AFOs) on clinical outcomes in stroke survivors.

**Methods:**

PubMed, Scopus, Web of Science, Embase, ProQuest, CENTRAL, REHABDATA, and PEDro were searched. The research team applied A Measurement Tool to Assess Systematic Reviews 2 (AMSTAR‐2) tool and Preferred Reporting Items for Systematic Reviews and Meta‐analyses (PRISMA) checklist for evaluating the reporting and methodological quality, respectively, and the ROBIS tool was used to evaluate the risk of bias (RoB) in the included reviews. The quality of the evidence was also judged using the (Grades of Recommendation, Assessment, Development and Evaluation) GRADE method.

**Results:**

In final, 14 SRs/MAs met inclusion criteria. Evaluation of methodological quality using the AMSTAR‐2 tool demonstrated that the overall quality of included reviews was mostly “critically low” or “low,” except for two studies that were “high.” In addition, the findings showed that the mean score of the reporting quality of the included reviews based on the PRISMA criteria was 24.9, down from 42. In accordance with the overall evaluation applying the ROBIS tool, 14.3% of the review studies were evaluated as high RoB, 64.3% were evaluated as unclear RoB, and 21.4% were evaluated as low RoB. Regarding the level of evidence quality, the GRADE results indicated that the evidence quality of the included reviews was unsatisfactory.

**Conclusion:**

This study showed that although the reporting quality of recently published SR/MAs evaluating the clinical effects of AFOs in stroke survivors was moderate, the methodological quality of almost all reviews was suboptimal. Therefore, reviewers must consider a number of criteria in designing, conducting, and reporting their studies to move toward transparent and conclusive results.

## 1 INTRODUCTION

Stroke is a major cause of death and disability in the world, with a high rate of chronic disability in survivors.^1^ Over the past three decades, the annual number of strokes has increased substantially, and its burden is likely to increase globally, particularly in low‐income countries.^2^ Rehabilitation measures are taken to improve the functional outcomes of stroke survivors. An orthosis is defined as a device aimed at correcting and supporting the structure and function of the musculoskeletal system.^3^ Ankle–foot orthoses (AFOs) are among the most commonly used rehabilitation options. AFOs are used to stabilize the foot and ankle during the stance phase, keeping the toes up while walking, and allowing for heel strike. Thus, AFOs are used as a rehabilitation intervention to improve the gait of stroke patients.^4^

Until now, many trials have studied the effects of AFOs on clinical outcomes in stroke survivors, and their data have been analyzed by relevant systematic reviews (SRs) and meta‐analyses (MAs). SRs are considered the gold standard for evidence used to assess the effectiveness of an intervention,^5^ and high‐quality SRs play an important role in evidence‐based clinical decision‐making for practitioners. Since some published SRs and MAs are not of high quality, precautions should be taken when using their findings as valid results. In fact, it has been stated that the large majority of SRs and MAs in biomedicine are likely redundant, misleading, and/or conflicted.^6^

Given the importance of SRs and MAs for practitioners, researchers, and policymakers, it is essential to assess them to ensure robust methodology and reliable results before applying them. In fact, some bias caused by a range of factors (e.g., reporting and methodological) can affect the validity and reliability of findings from SRs/MAs.^7^ Currently, no methodological study has evaluated the quality of SRs assessing the effects of AFOs and identified the main factors affecting the quality of such studies. Various tools and checklists have been developed in recent years to evaluate various aspects of these studies. This methodological SR is the first study to appraise the methodological and reporting quality of the recently published SR/MAs assessing the effects of AFOs on the clinical outcomes of stroke survivors.

## 2 METHODS

This study has been written in accordance with the Preferred Reporting Items for Systematic Reviews and Meta‐analyses (PRISMA) criteria.^8^ Furthermore, the research team considered the criteria of A Measurement Tool to Assess Systematic Reviews 2 (AMSTAR‐2) to enhance the reporting quality.^9^ The protocol of this study was already reviewed and registered with the Iran National Committee for Biomedical Research (Registration number: IR.SUMS.REC.1401.241, available here: https://b2n.ir/s72067).

### 2.1 Search strategy

Three components of PICO‐S (population, intervention, comparison, outcome, and study design), including population (stroke patients), intervention (AFO), and study design (SR/MA), were adhered to when developing search strings. The research team tried to identify the most relevant terms using Medical Subject Headings (MeSH) and Emtree thesauruses. In addition, a free‐text approach and contacting relevant experts were used to find more terms. The search string was first created for the PubMed database and then adapted for other electronic databases. Keywords such as “ankle–foot orthosis,” “ankle foot orthosis,” “ankle–foot orthoses,” “ankle foot orthoses,” “Foot orthotic devices,” “stroke, strokes,” “Cerebrovascular Accident,” “Cerebrovascular Accidents,” “CVA,” “Cerebrovascular Apoplexy,” “systematic review,” “review,” “meta analysis,” “meta‐analysis,” and “meta analyses” were considered to develop search strings (Supporting Information: Table 1).

PubMed, Scopus, WoS (Web of Science), Embase (Excerpta Medica Database), ProQuest, CENTRAL (Cochrane Central Register of Controlled Trials), REHABDATA, and PEDro (Physiotherapy Evidence Database) were searched from January 1, 2000, to the end of December 2021. The search process was conducted by the first author, and no language restriction was adopted. In addition, Google Scholar, OpenGrey, Microsoft Academic, and F1000Research were searched to find any unpublished studies. Further, the research team manually assessed the reference lists of relevant studies and also key journals (including *Disability and Rehabilitation*, *Clinical Rehabilitation*, *Prosthetics and Orthotics International*, *Journal of Prosthetics and Orthotics*, *Archives of Physical Medicine and Rehabilitation*, *PLoS ONE*, *Stroke, Gait & Posture*, etc.).

All of the initial records identified through searching electronic databases were imported into Endnote X8 software (Thomson Reuters) to withdraw the duplicates, and then the remaining records were screened based on the title and abstract by two reviewers (S. Sh. and M. J.) independently. In the next steps, the same two reviewers independently began assessing the full text of potential studies against inclusion and exclusion criteria to retrieve the final studies. Any conflict between two reviewers was resolved by discussion, and if required, the assistance of the third reviewer (P. M.) was used.

### 2.2 Eligibility criteria

The current study included SRs and MAs who were evaluating the effects of AFO on clinical outcomes in stroke patients. The clinical outcomes of interest included gait parameters (walking speed, cadence, step length, stride length), balance, the Timed Up and Go test, the Functional Ambulation Category, energy expenditure, and hip/ankle kinematics. The eligible SR/MAs had at least evaluated the effects of AFO as one of the main primary aims in the following comparisons: (1) AFO versus no intervention; (2) AFO versus another intervention; (3) one type of AFO versus another type of AFO; (4) AFO combined with another intervention versus another intervention or only AFO. We excluded other types of review studies (including scoping reviews, critical reviews, umbrella reviews, etc.). Protocol studies, conference abstracts, SR/MA of animals, and studies lacking full text were also excluded.

### 2.3 Data extraction

This stage was performed by two reviewers (S. Sh. and P. M.) independently. As in the previous steps, any conflicts were resolved through discussion and the assistance of the third reviewer (P. M.). The following data were extracted from the included SR/MAs: (1) first author; (2) publication year; (3) country; (4) journal name; (5) impact factor of the journal; (6) type of study design; (7) population; (8) intervention and comparison arms; (9) number of included studies; (10) protocol registration details; (11) outcome(s); (12) risk of bias (RoB) assessment tool; (13) summary of findings; and (14) funding source.

### 2.4 Reporting and methodological quality assessment

The research team applied the PRISMA checklist and AMSTAR‐2 tool for evaluating the reporting and methodological quality, respectively. PRISMA, as a reporting guideline, consists of 7 sections (title, abstract, introduction, methods, results, discussion, and other information) and 27 items. Each item was assessed as “yes” (full reports), “partial yes” (partial reports), or “no” (no reports).^8^ The adherence of each item is revealed as a ratio. The AMSTAR‐2 tool, which was published recently, consists of 16 items, and each item could be judged as “yes,” “partial yes,” or “no.” The final rating of quality can be presented as “high,” “moderate,” “low,” and “critically low.”^9^ There are seven critical domains (items 2, 4, 7, 9, 11, 13, and 15) in the AMSTAR‐2 tool that affect the overall rating. The research team rated the overall methodological quality in accordance with the AMSAR 2 tool as follows: (1) “high”: if there is not any major concern regarding the critical domains but up to three flaws identified regarding other domains; (2) moderate: if there are more than three flaws identified regarding noncritical domains; (3) low: if there is a major flaw regarding a critical domain; and (4) critically low: if there are more than one major flaw in critical domains.^9^, ^10^

Forest plots were used to indicate the proportions of included studies meeting complete adherence to PRISMA and AMSTAR‐2 criteria. To establish such plots, two‐sided 95% confidence intervals (CIs) of percentages were considered using the Wilson Score method. Statistical analyses were conducted via Stata 14.0 software (StataCorp LP).

### 2.5 RoB assessment

ROBIS is a validated tool to evaluate the RoB within SR studies. This tool consists of three phases: (a) assessing relevance; (b) identifying concerns with the review process; and (c) judging RoB. The first phase is optional, and we did not consider it within the present study. Based on this tool, the overall level of RoB can be rated as “low,” “high,” or “unclear.”^10^, ^11^ In final, the Grading of Recommendations Assessment, Development, and Evaluation^12^ system was used to assess the quality of the evidence. Six criteria, including RoB, publication bias, imprecision, indirectness, effect size, and inconsistency, were applied to determine the level of quality as high, moderate, low, and very low.^12^, ^13^ Based on the available information, we developed the Grades of Recommendation, Assessment, Development and Evaluation (GRADE) profile for the walking speed outcome. Two reviewers independently conducted the aforementioned steps, and in case of any disagreement, the assistance of a third reviewer (P. M.) was used.

## 3 RESULTS

**Figure 1:**
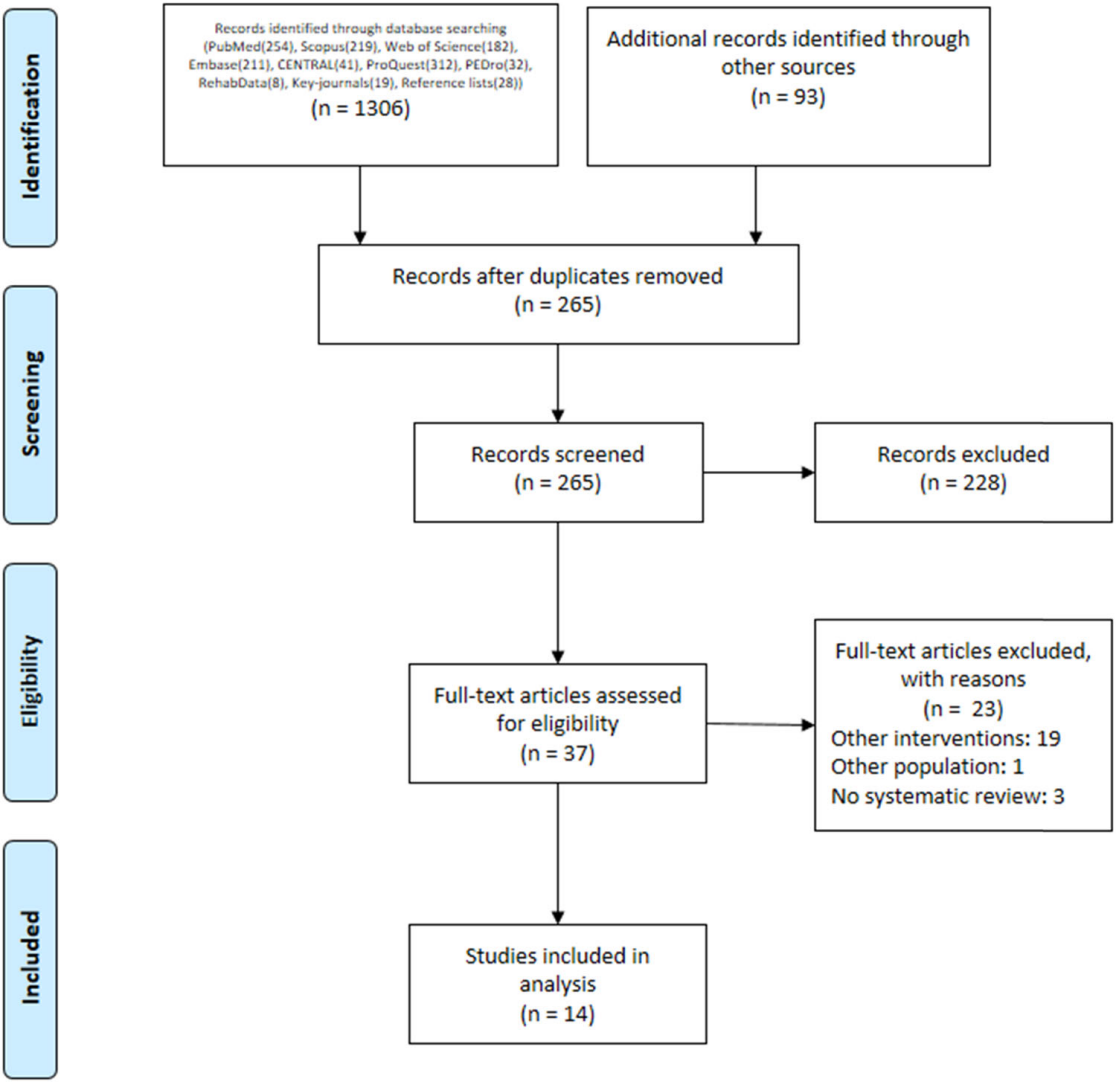
The selection process of studies included in this review.

After the initial search, 1399 studies were found, and after removing duplicates, 265 remained, which were screened by two reviewers independently based on the title and abstract. In the next step, 37 potentially relevant studies were then identified and evaluated by the two reviewers independently based on the full text. Finally, 14 studies^4^ ^,^ ^14^, ^15^, ^16^, ^17^, ^18^, ^19^, ^20^, ^21^, ^22^, ^23^, ^24^, ^25^, ^26^ were selected by the research team to enter the study (Figure 1). In the final step, 23 studies were excluded, along with the reasons for exclusion, which are listed in Supporting Information: Table 2.

### 3.1 Characteristics of included reviews

Table 1 summarizes the characteristics of the final included reviews. The 14 review studies consisted of 8 SRs with meta‐analysis,^4^, ^14^, ^16^, ^18^, ^19^, ^23^, ^24^, ^25^ 4 SRs,^15^, ^17^, ^22^, ^26^ and 2 meta‐analyses.^20^, ^21^ Based on the country of origin for the corresponding author, 37.7% of the reviews were from the United Kingdom, 28.6% were from Iran, 14.2% were from Brazil, 7.1% were from the Republic of Korea, 7.1% were from Spain, and 7.1% were from Japan. The most common outcomes assessed in the included reviews were walking speed, balance, energy consumption, gait parameters, and mobility. The Cochrane RoB Tool, the Downs and Black Checklist, and the PEDro Scale were the common RoB assessment tools that were applied in the included reviews. However, the JBI critical appraisal checklist and Jadad scale were applied in two reviews.^22^, ^23^ Notably, only two studies^18^, ^23^ stated their source of funding, and two studies^4^, ^24^ stated that they did not have financial support. As shown in Supporting Information: Figure 1, the largest number of studies were published in 2013, 2020, and 2021, with three review studies being published each year. In addition, with the exception of one study,^26^ other studies have been published in peer‐reviewed journals with an impact factor of 2–4.5.

**Table 1:** Characteristics of included reviews.

References	Country	Journal	Type	Population	Intervention	Comparison	Outcome(s)	Number of studies included	Number of study participants	Risk‐of‐bias assessment tool	Summary of findings of review	Funding source
Choo and Chang^18^	Republic of Korea	Scientific Reports	SR and MA	Stroke patients	AFO	Without AFO	Walking speed Cadence Step length Stride length TUG test FAC score Ankle sagittal plane angle Knee sagittal plane angle	19	434	Cochrane Risk of Bias Tool	The FAC score showed the most significant improvement, and stride time showed the lowest improvement. AFO improves walking speed, cadence, step length, and stride length, particularly in patients with stroke. AFO is considered beneficial in enhancing gait stability and ambulatory ability.	National Research Foundation of Korea Grant funded by the Korean government
Daryabor et al.^16^	Iran	Disability and Rehabilitation	SR and MA	Stroke patients	AFO	Without AFO	Balance Walking speed Functional mobility Motricity Index	30	669	Downs and Black checklist	AFO can improve functional performance and ambulation in survivors of strokes and that an AFO is more effective on functional outcomes with long‐term adaptation. Wearing an AFO in rehabilitation care during the subacute phase may have beneficial effects on clinical outcomes measured in individuals with a history of stroke.	N/A
Daryabor et al.^17^	Iran	Disability and Rehabilitation	SR	Stroke patients	AFO	Without AFO Other AFO designs	Energy consumption Energy cost PCI Vertical COM trajectory Mechanical work	15	195	Downs and Black checklist	Although the studies were somewhat weak in scientific rigor and had moderate risks of bias, this review has demonstrated that an AFO can make an immediate, short‐time improvement in the energy cost of walking, while the AFO is worn.	N/A
Daryabor et al.^15^	Iran	Gait & Posture	SR	Stroke patients	AFO	Without AFO Other AFO designs	Ankle kinematics Knee kinematics Hip kinematics Kinetic (moment, power, and GRF)	27	336	Downs and Black checklist	Using an AFO can make an immediate improvement in walking. Although nonarticulated passive AFOs (in particular, RAFO) improve pathological gait of hemiplegia to some extent, it limits some motions with functional benefits. The articulated passive AFO with plantarflexion stops can prevent drop‐foot successfully by providing dorsiflexion assisting force; these AFOs also inhibit other normal movements of the ankle.	N/A
Ferreira et al.^26^	Brazil	The Journal of Physical Therapy Science	SR	Stroke patients	AFO	No intervention Different interventions	Gait velocity Cadence	13	315	PEDro scale	All types of AFO resulted in a significant improvement in gait velocity compared to a control group without the use of an AFO. However, divergent results were reported for cadence with and without the use of an AFO, as some studies report an improvement in this variable and others report no significant improvement.	N/A
Hollands et al.^23^	United Kingdom	Gait & Posture	SR and MA	Stroke patients	AFO (among other interventions)	Without AFO	Gait speed Self‐selected gait symmetry	4	Not specified	JBI critical appraisal checklist	The effect of AFO on gait symmetry and gait speed was trivial.	Stroke Association
Nascimento et al.^19^	Brazil	Physiotherapy	SR and MA	Stroke patients	AFO	Without AFO FES Placebo	Walking speed Balance	11	1135	PEDro scale	Moderate‐quality evidence indicated that both AFO and functional electrical stimulation improve walking speed after stroke. In addition, moderate‐to‐high‐quality evidence indicated no superiority of AFO, in comparison with functional electrical stimulation, for improving walking speed or balance.	N/A
Guerra Padilla et al.^22^	Spain	Neurología	SR	Stroke patients	AFO	Without AFO other interventions	Walking speed Balance	10	289	Jadad scale	An AFO may be prescribed soon after a stroke event to improve certain gait parameters, such as velocity and cadence. It may also offer the patient greater stability, which will increase his/her self‐confidence and postural control by enabling participation in more activities.	N/A
Prenton et al.^21^	United Kingdom	Journal of Rehabilitation Medicine	MA	Stroke patients	AFO	FES	Walking speed Fugl‐Meyer Kinematics EMG Activity	6	450	Cochrane Risk of Bias Tool	Functional electrical stimulation and AFOs have an equally positive therapeutic effect on walking speed in nonprogressive central nervous system diagnoses.	N/A
Prenton et al.^20^	United Kingdom	Journal of Rehabilitation Medicine	MA	Stroke patients	AFO	FES	Walking speed Functional exercise capacity Perceived mobility	5	815	Cochrane Risk of Bias Tool	In contrast to assumptions that predict FES superiority, AFOs have equally positive combined‐orthotic effects as FES on key walking measures for foot‐drop caused by stroke.	N/A
Shahabi et al.^4^	Iran	Clinical Rehabilitation	SR and MA	Stroke patients	AFO	Without AFO Other interventions	Walking speed	14	1186	Cochrane Risk of Bias Tool	There is inadequate evidence that an AFO is associated with a detectable sustained improvement in walking speed in patients who have suffered a stroke.	No financial support
Tyson et al.^24^	United Kingdom	Clinical Rehabilitation	SR and MA	Stroke patients	AFO	Without AFO	Ankle kinematics Hip kinematics Kinetics Energy cost Energy expenditure	20	314	PEDro scale	An AFO can improve the ankle and knee kinematics, kinetics and energy cost of walking in stroke survivors.	No specific financial support
Tyson et al.^25^	United Kingdom	Archives of Physical Medicine and Rehabilitation	SR and MA	Stroke patients	AFO	Without AFO	Mobility TUG test Walking speed Step/stride length Balance Postural sway Weight distribution	13	334	Cochrane Risk of Bias Tool	An AFO can improve walking and balance after stroke, but only the immediate effects have been examined.	N/A
Wada et al.^14^	Japan	PM&R: The Journal of Injury, Function and Rehabilitation	SR and MA	Stroke patients	AFO	Without AFO	Walking speed Functional mobility Quality of life Activity	14	282	Cochrane Risk of Bias Tool	AFO improved ankle kinematics and walking ability in the short term; nonetheless, the evidence was characterized by a low degree of certainty.	N/A

Abbreviations: AFO, ankle–foot orthosis; CoM, center of mass; FAC, functional ambulation categories; EMG, electromyography; FES, functional electrical stimulation; GRF, ground reaction force; JBI, Joanna Briggs Institute; MA, meta‐analysis; N/A, not mention; PCI, physiological cost index; SR, systematic review; TUG, timed up and go.

### 3.2 Reporting and methodological quality

**Figure 2:**
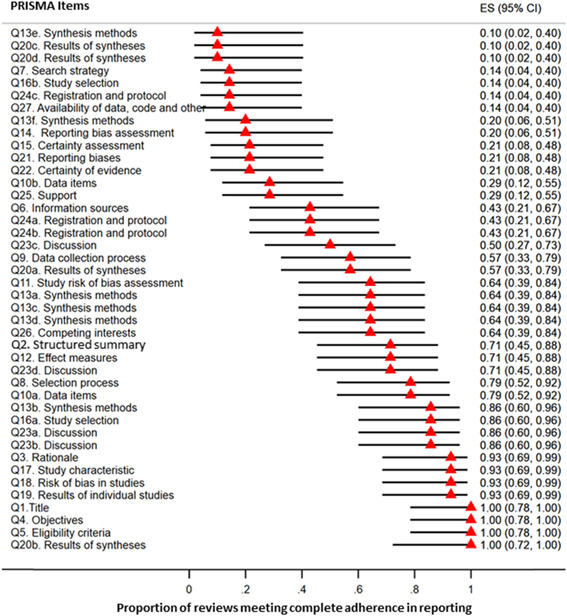
Forest plot of the proportion of included studies meeting complete adherence to PRISMA criteria. The results have been showed by ES and associated 95% CI. CI, confidence interval; ES, effect size; PRISMA, preferred reporting items for systematic reviews and meta‐analyses.

Table 2 shows the results of reporting quality assessment in accordance with the PRISMA criteria. As a whole, the mean adherence of included reviews to the PRISMA criteria was 24.9 from 42 (median: 26.5, interquartile range [IQR]: 8.5). Based on the results, only four items (Q1, Q4, Q5, and Q20b) of the PRISMA statement were adhered to by all studies. Specifically, the seventh item in this reporting guideline, which evaluates the search strategy, was not well considered by review studies as the adherence rate was 14%. In addition, only one study^4^ conducted subgroup analysis to find the causes of heterogeneity among the included studies (Q13e). The results revealed that sensitivity analysis, evaluation of reporting bias, and the approach of evaluating the certainty of the findings have rarely been considered in both the methods and results sections of the review studies (Q13f, Q14, and Q15). Although it has recently been recommended that a list of excluded studies be created along with the exclusion reason following the evaluation of studies based on the full text against interested criteria (Q16b), this recommendation was only considered in a limited number of included studies.^4^, ^14^, ^16^, ^17^ Regarding protocol registration, only six of the included studies^4^, ^14^, ^16^, ^19^, ^20^, ^21^, ^24^ (43%) had previously registered and published their protocols. Furthermore, just two review studies^4^, ^14^ (14%) explained subsequent amendments made to the primary registered protocol. Figure 2 includes a forest plot, which reveals the proportions with associated 95% CIs of included reviews meeting total adherence to PRISMA criteria.

**Table 2:** Results of the PRISMA checklist.

Section/topic	Items	Choo and Chang^18^	Daryabor et al.^16^	Daryabor et al.^17^	Daryabor et al.^15^	Ferreira et al.^26^	Hollands et al.^23^	Nascimento et al.^19^	Guerra Padilla et al.^22^	Prenton et al.^21^	Prenton et al.^20^	Shahabi et al.^4^	Tyson et al. (2013a)	Tyson et al. (2013b)	Wada et al.^14^
Title	Q1. Title	Y	Y	Y	Y	Y	Y	Y	Y	Y	Y	Y	Y	Y	Y
Abstract	Q2. Structured summary	Y	Y	Y	PY	PY	PY	Y	PY	Y	Y	Y	Y	Y	Y
Introduction	Q3. Rationale	Y	Y	Y	Y	N	Y	Y	Y	Y	Y	Y	Y	Y	Y
	Q4. Objectives	Y	Y	Y	Y	Y	Y	Y	Y	Y	Y	Y	Y	Y	Y
Methods	Q5. Eligibility criteria	Y	Y	Y	Y	Y	Y	Y	Y	Y	Y	Y	Y	Y	Y
	Q6. Information sources	PY	PY	PY	PY	PY	Y	Y	PY	PY	PY	Y	Y	Y	Y
	Q7. Search strategy	N	PY	PY	PY	N	N	Y	N	N	N	Y	N	N	PY
	Q8. Selection process	Y	Y	Y	PY	N	Y	Y	N	Y	Y	Y	Y	Y	Y
	Q9. Data collection process	PY	Y	Y	PY	N	Y	Y	N	Y	Y	Y	Y	PY	PY
	Q10a. Data items	PY	Y	Y	Y	N	Y	Y	Y	Y	Y	Y	PY	Y	Y
	Q10b. Data items	PY	PY	Y	N	N	Y	Y	PY	PY	PY	PY	N	PY	Y
	Q11. Study risk of bias assessment	Y	PY	PY	PY	PY	Y	Y	Y	Y	N	Y	Y	Y	Y
	Q12. Effect measures	Y	Y	N	N	N	Y	Y	N	Y	Y	Y	Y	Y	Y
	Q13a. Synthesis methods	Y	Y	Y	PY	N	Y	Y	N	Y	PY	Y	PY	Y	Y
	Q13b. Synthesis methods	Y	Y	Y	Y	N	Y	Y	N	Y	Y	Y	Y	Y	Y
	Q13c. Synthesis methods	Y	Y	N	N	N	Y	Y	N	Y	Y	Y	Y	N	Y
	Q13d. Synthesis methods	Y	Y	N	N	N	Y	Y	N	PY	Y	Y	Y	Y	Y
	Q13e. Synthesis methods	N	N	N/A	N/A	N/A	N	N	N/A	N	N	Y	N	N	N
	Q13f. Synthesis methods	N	N	N/A	N/A	N/A	N	N	N/A	Y	N	Y	N	N	N
	Q14. Reporting bias assessment	N	Y	N/A	N/A	N/A	N	N	N/A	N	N	Y	N	N	N
	Q15. Certainty assessment	N	N	N	N	N	N	Y	N	N	N	Y	N	N	Y
Results	Q16a. Study selection	Y	Y	Y	Y	PY	Y	Y	N	Y	Y	Y	Y	Y	Y
	Q16b. Study selection	N	N	N	N	N	N	N	N	PY	N	Y	PY	N	Y
	Q17. Study characteristic	Y	Y	Y	Y	N	Y	Y	Y	Y	Y	Y	Y	Y	Y
	Q18. Risk of bias in studies	Y	Y	Y	Y	Y	Y	Y	PY	Y	Y	Y	Y	Y	Y
	Q19. Results of individual studies	Y	Y	Y	Y	N	Y	Y	Y	Y	Y	Y	Y	Y	Y
	Q20a. Results of syntheses	PY	PY	PY	PY	N	Y	Y	Y	Y	Y	PY	Y	Y	Y
	Q20b. Results of syntheses	Y	Y	N/A	N/A	N/A	Y	Y	N/A	Y	Y	Y	Y	Y	Y
	Q20c. Results of syntheses	N	N	N/A	N/A	N/A	N	N	N/A	N	N	Y	N	N	N
	Q20d. Results of syntheses	N	N	N/A	N/A	N/A	N	N	N/A	N	N	Y	N	N	N
	Q21. Reporting biases	Y	Y	N	N	N	N	N	N	N	N	Y	N	N	N
	Q22. Certainty of evidence	N	N	N	N	N	N	Y	N	N	N	Y	N	N	Y
Discussion	Q23a. Discussion	Y	Y	Y	Y	Y	PY	Y	Y	Y	Y	Y	Y	PY	Y
	Q23b. Discussion	Y	Y	Y	Y	PY	Y	Y	Y	Y	Y	Y	Y	Y	N
	Q23c. Discussion	Y	N	N	N	PY	Y	Y	PY	PY	Y	N	Y	Y	Y
	Q23d. Discussion	Y	Y	Y	Y	N	Y	N	Y	PY	Y	Y	Y	Y	N
Other information	Q24a. Registration and protocol	N	Y	N	N	N	N	Y	N	Y	Y	Y	N	N	Y
	Q24b. Registration and protocol	N	Y	N	N	N	N	Y	N	Y	Y	Y	N	N	Y
	Q24c. Registration and protocol	N	N	N	N	N	N	N	N	N	N	Y	N	N	Y
	Q25. Support	Y	N	N	N	N	Y	N	N	N	N	Y	Y	N	N
	Q26. Competing interests	Y	Y	Y	N	N	N	N	Y	Y	Y	Y	Y	N	Y
	Q27. Availability of data, code and other materials	Y	Y	N	N	N	N	N	N	N	N	N	N	N	N
Total score	27.5	29.5	21	17	8	26	30	15.5	28	26.5	39	26.5	23.5	31	

Abbreviations: N, no; N/A, not applicable; PRISMA, Preferred Reporting Items for Systematic Reviews and Meta‐analyses; PY, partial yes; Y, yes.

**Figure 3:**
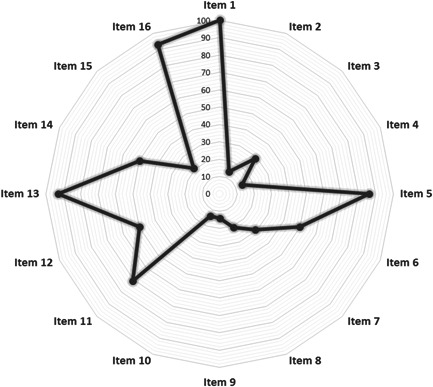
Radar chart of AMSTAR‐2 items. This chart reveals the adherence of included studies to each item of AMSTAR‐2 tool. AMSTAR‐2, a measurement tool to assess systematic reviews 2.

**Figure 4:**
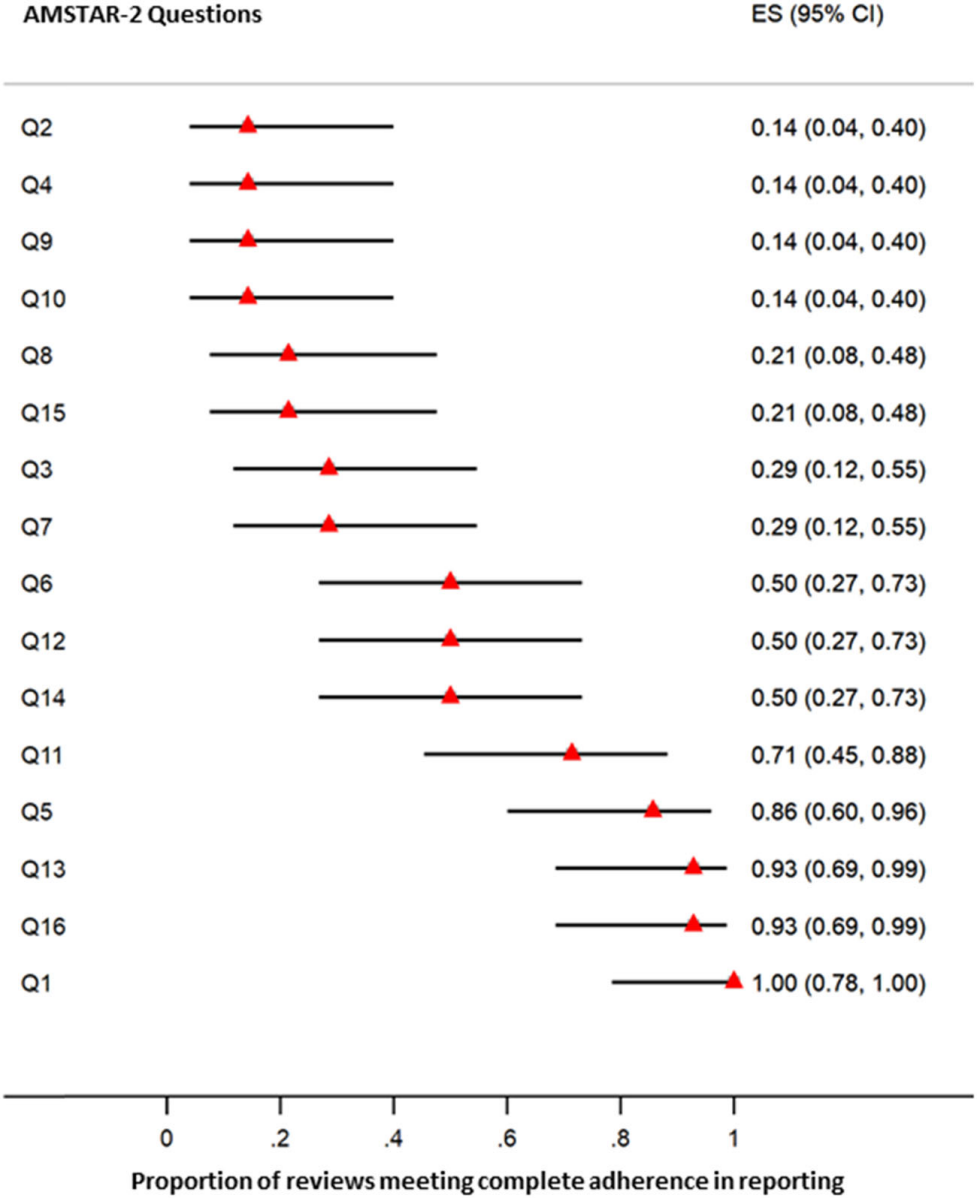
Forest plot of the proportion of included studies meeting complete adherence to AMSTAR‐2 items. The results have been showed by ES and associated 95% CI. AMSTAR‐2, a measurement tool to assess systematic reviews 2; CI, confidence interval; ES, effect size.

Evaluation of methodological quality using the AMSTAR‐2 tool demonstrated that the overall quality of included reviews was mostly “critically low” or “low” (further details can be found in Table 3 and Figure 3). Interestingly, only the quality of the two studies^4^, ^16^ was high in accordance with the aforementioned tool. As described previously, only a small number of review studies^4^, ^14^, ^16^, ^19^, ^20^, ^21^, ^24^ had preregistered their protocols, which is why many of the included studies did not meet the criteria of the second item of the AMSTAR‐2 tool. Furthermore, except for two studies,^4^, ^14^ all included reviews were judged “partial yes” based on the fourth item (a comprehensive literature search strategy). Indeed, the inadequate search of gray literature, registry databases, and reference list of included and related studies are the most important flaws in this evaluation. Regarding item 10, only two review studies^4^, ^14^ have considered the funding source of primary studies, a topic that has recently been raised by reporting guidelines to reduce agenda bias. Although the RoB and quality status of primary studies were considered by all review studies (item 13), only 50% of them discussed the potential for heterogeneity in their results (item 14). Among SR/MA studies, just three studies^4^, ^16^, ^18^ (33.3%) conducted publication bias (small study effect) assessment using a statistical method or funnel plot (item 15). Figure 4 indicates the proportions with associated 95% CIs of included reviews meeting total adherence to AMSTAR‐2 items.

**Table 3:** Results of the AMSTAR‐2 assessments.

References	AMSTAR‐2																Quality
	Q1	Q2	Q3	Q4	Q5	Q6	Q7	Q8	Q9	Q10	Q11	Q12	Q13	Q14	Q15	Q16	
Choo & Chang^16^	Y	N	N	PY	Y	Y	N	PY	Y	N	Y	N	Y	Y	Y	Y	Critically low
Daryabor et al.^17^	Y	Y	Y	PY	Y	Y	Y	Y	PY	N	Y	Y	Y	Y	Y	Y	High
Daryabor et al.^18^	Y	N	N	PY	Y	N	Y	Y	PY	N	N	N	Y	N	N	Y	Low
Daryabor et al.^19^	Y	N	N	PY	Y	N	N	PY	PY	N	N	N	Y	N	N	Y	Critically low
Ferreira et al.^15^	Y	N	N	PY	N	N	N	PY	PY	N	N	N	N	N	N	N	Critically low
Hollands et al.^20^	Y	N	N	PY	Y	Y	N	PY	PY	N	Y	N	Y	Y	N	Y	Critically low
Nascimento et al.^21^	Y	PY	N	PY	Y	Y	N	Y	PY	N	Y	Y	Y	Y	N	Y	Critically low
Padilla et al. (2014)	Y	N	N	PY	N	N	N	PY	PY	N	N	Y	Y	N	N	Y	Critically low
Prenton et al.^22^	Y	PY	N	PY	Y	N	N	PY	PY	N	Y	Y	Y	N	N	Y	Critically low
Prenton et al.^23^	Y	PY	N	PY	Y	N	N	PY	PY	N	Y	Y	Y	N	N	Y	Critically low
Shahabi et al.^4^	Y	Y	N	Y	Y	Y	Y	PY	Y	Y	Y	Y	Y	Y	Y	Y	High
Tyson et al. (2013a)	Y	N	Y	PY	Y	Y	N	PY	PY	N	Y	Y	Y	Y	N	Y	Critically low
Tyson et al. (2013b)	Y	N	Y	Y	Y	N	N	PY	PY	N	Y	N	Y	Y	N	Y	Critically low
Wada et al.^14^	Y	Y	Y	PY	Y	Y	Y	PY	PY	Y	Y	N	Y	N	N	Y	Low

*Note*: Highlighted columns are the critical questions of AMSTAR‐2 checklist.

Abbreviations: Yes, Y; Partial Yes, PY; No, N.

### 3.3 RoB assessment

**Figure 5:**
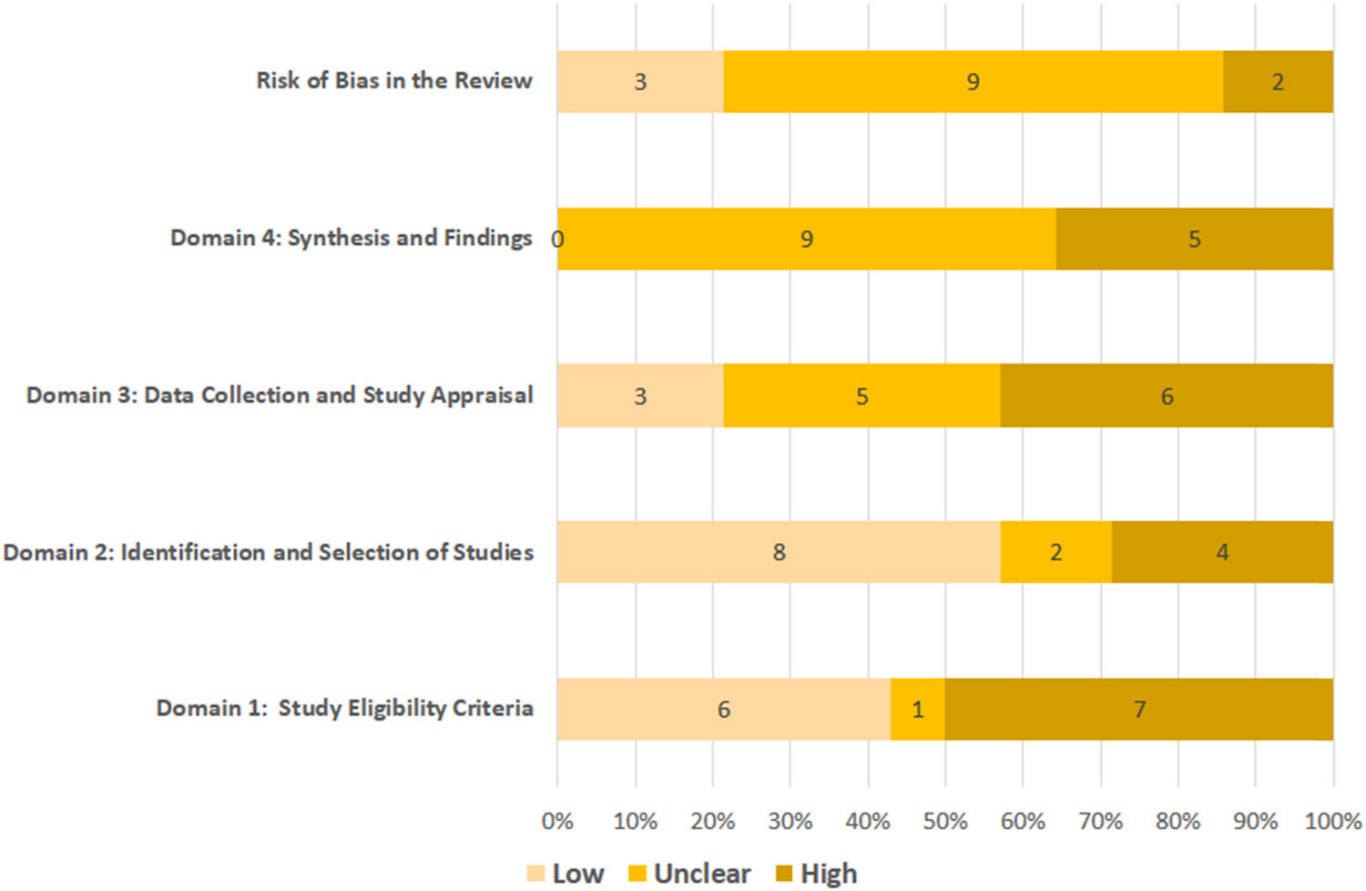
The results of risk of bias assessment of included studies using ROBIS tool. ROBIS is a tool to evaluate the risk of bias in systematic reviews.

Figure 5 demonstrates the summaries of ROBIS scores. Based on the ROBIS criteria, 50% of included studies had a low RoB level in the domain of “study eligibility criteria.” However, 42.8% of studies had a high RoB level, and 7.2% of studies had an unclear RoB level in this domain. Regarding the domain of “identification and selection of studies,” 57.1% of included studies had a low RoB level. Nonetheless, 78.5% of review studies had high or unclear RoB level in the domain of “data collection and study appraisal.” In addition, all review studies had a high or unclear RoB level regarding the domain of “synthesis and findings.” In accordance with the overall evaluation applying the ROBIS tool, 14.3% of the review studies were evaluated as high RoB, 64.3% were evaluated as unclear RoB, and 21.4% were evaluated as low RoB. A summary of the scores for each domain for the included reviews can be seen in Supporting Information: Table 3.

### 3.4 Quality of evidence

Table 4 shows the summary of findings for the walking speed outcome in accordance with the GRADE approach. Based on the findings, the quality of the evidence in three included studies^14^, ^19^, ^23^ was very low, the quality of the evidence in three included studies^4^, ^20^, ^21^ was low, and the quality of the evidence in three included studies^4^, ^18^, ^25^ was moderate.

**Table 4:** Summary of findings.

References		No. of studies	Study design of include studies	Risk of bias	Inconsistency	Indirectness	Imprecision	Publication bias	Effect size	GRADE quality
Effects of AFO on walking speed										
Choo and Chang^18^	AFO vs. without AFO	13	RCT/CT	−1^a^	0	0	0	0	0	Moderate
Hollands et al.^23^	AFO vs. without AFO	3	CT	0	0	0	−2^b^	−1^c^	0	Very low
Nascimento et al.^19^	AFO vs. without AFO	2	RCT	−1	0	0	−2	−1	0	Very low
Shahabi et al.^4^	AFO vs. without AFO	4	RCT	−1	0	0	−1^d^	0	0	Low
Tyson et al.^25^	AFO vs. without AFO	11	RCT/CT	0	0	0	0	−1	0	Moderate
Wada et al.^14^	AFO vs. without AFO	15	RCT	−1	−1^e^	0	0	−1	0	Very low
Nascimento et al.^19^	AFO vs. FES	4	RCT	−1	−1	0	0	−1	0	Very low
Prenton et al.^21^	AFO vs. FES	5	RCT	−1	0	0	0	−1	0	Low
Prenton et al.^20^	AFO vs. FES	5	RCT	−1	0	0	0	−1	0	Low
Shahabi et al.^4^	AFO vs. FES	8	RCT	−1	0	0	0	0	0	Moderate

Abbreviations: AFO, ankle–foot orthoses; GRADE, Grades of Recommendation, Assessment, Development and Evaluation; RCT, randomized controlled trial.

^a^ Downgraded one level as the moderate risk of bias.

^b^ Downgraded two level as the number of included studies was very low (<3).

^c^ Downgraded one level as the publication bias has not evaluated.

^d^ Downgraded one level as the number of included studies was low (<5).

^e^ Downgraded one level as the *I* ^2^ was >50%.

## 4 DISCUSSION

SRs are placed at the pinnacle of the pyramid of evidence and are used to address questions and provide evidence for interventions. This is the first methodological SR evaluating the quality of SR/MAs assessing the effects of AFO on clinical outcomes in stroke patients. This study included 14 SR/MAs from six countries, based on the affiliation of the corresponding authors. None of the reviews examined came from the United States or China, the two countries that have produced the most scientific documents in the last two decades.^27^ Six studies were published in the two most recent years (2020–2021), and only three studies were published in journals whose main scopes were other than rehabilitation. Walking speed was the most commonly assessed clinical outcome, presumably due to its simplicity and functional significance.^28^

Six studies considered the Cochrane RoB Tool. The PEDro scale was used by three studies. This scale is an old tool, with its last modification in 1999.^29^ It is primarily used for assessing the RoB in RCTs in the PEDro database. This database contains trials, reviews, and guidelines evaluating physical therapy interventions. Compared to the PEDro scale, the Cochrane RoB Tool considers additional sources of bias, such as sequence generation, allocation concealment, selective result reporting, blinding, and incomplete result data.^30^ Disagreements between the quality assessed according to the Cochrane and PEDro criteria have been demonstrated, leading to different sets of trials of adequate quality.^31^ There is moderate agreement for three of the six PEDro and Cochrane items that evaluate similar constructs, and they can't be used interchangeably.^32^ Daryabor et al. used the Downs and Black checklist originally introduced in 1998.^33^ This numerical rating scale was shown to fail to identify studies with increased RoB.^34^ A cross‐sectional analysis of SR protocols on health interventions registered in PROSPERO showed that the Cochrane RoB tool was the most frequently used appraisal tool for RCTs; however, nonrandomized trials were assessed by widely varied tools.^35^

In this study, the Joanna Briggs Institute critical appraisal checklist, used by one of our studies, was among the least frequently used appraisal tools. The Jadad scale, introduced in 1996, was used in one of our studies. Due to its inability to detect some serious biases, it has been recommended to discontinue its use for evaluating the quality of trials.^36^ The need for a gold standard tool, especially for nonrandomized trials, is felt by authors conducting rehabilitation‐related studies. Sponsorship bias has long been recognized in clinical trials.^37^ Similarly, PRISMA requests a description of sources of support and the role of funders or sponsors in the review. Most of the included studies were silent on this item. Cochrane has taken precautions to minimize the financial ties of authors of SRs to industry.

According to the PRISMA checklist, the weak points of the (included) review studies seem to be mostly occulted in search strategy, synthesis methods (methods to explore heterogeneity and sensitivity analysis), study selection both in method and results, results of synthesis (heterogeneity and sensitivity), reporting bias assessment, and certainty of evidence. According to the AMSTAR‐2, the overall confidence in the results of the majority of the included studies (10 out of 14) was considered to be “critically low.” Although AMSTAR‐2 is an almost new assessment tool, and only a few studies have used it, our result was in line with similar studies in some other fields.^10^, ^38^, ^39^, ^40^, ^41^, ^42^ In the current study, there was a moderate association (Speraman's *ρ* = 0.582, *p* = 0.024) between PRISMA score and AMSTAR‐2 quality assessment.

SRs have been recommended to register their protocol, including major objectives, design features, and prospective analyses for the review at inception. The first advantage of protocol registration is to avoid duplications. Also, adherence to a predefined method may reduce the RoB in future results.^43^ Only half of the included studies had reported prospective registration of their protocol. Even those published in recent years, the remainders did not report preregistered protocol. Unsurprisingly, the two studies with high RoB based on ROBIS were among this group. However, most of the studies were categorized as unclear.

The study eligibility criteria are evaluated in domain 1 of phase 2 of ROBIS. This item ensures consistency in including the studies in a review. Assessment of this domain would be more accurate if review studies provided a registered document or profile before reporting the results and adhered to it while conducting the review. An SR is conducted to answer a clearly defined question. Based on the question, decisions are made regarding which primary studies should be included. Only half of the included studies were categorized as having low concerns regarding this item. That means it is very likely that some relevant studies have been excluded or that even inappropriate studies have been included. This can occur as a result of inappropriate restrictions or inconsistency in study selection. A trustworthy SR includes all qualified primary studies. This would be ascertained by making a deliberate effort to do a sensitive search to elicit all possible eligible studies. Four reviews were graded as high concern, which shows some eligible studies are likely to be missing from the review. Most of the reviews were graded as high concern in data collection and study appraisal domain. Errors in data collection, insufficiently available study characteristics, missed relevant results, inappropriate criteria to assess RoB, and errors in RoB assessment may have led to this result. All of the included reviews were classified as high‐risk or unclear regarding synthesis and findings. This result warns that the synthesis and findings of reviews are not free from bias. Biases may have come from different sources, like important between‐study variation, inadequacies in the methodology, and incompletely reported findings.

## 5 LIMITATIONS

The current study has a number of limitations. First, since the data extraction was unblinded, it could have been a potential source of bias in the expectations of reviewers about the quality of papers from high‐ranked journals. Second, the generalizability of our findings may be restricted as papers evaluating SR/MA studies evaluate the effects of AFO on stroke patients. Indeed, AFO is an integral part of rehabilitation for patients with cerebral palsy, and there are several SR/MA studies on the effectiveness of AFO in this field. Thus, it is suggested that future studies be conducted in this field.

## 6 CONCLUSION

This study showed that although the reporting quality of recently published SR/MAs evaluating the clinical effects of AFOs in stroke survivors was moderate, the methodological quality of almost all reviews was suboptimal because of the nonregistration of protocols, the lack of a comprehensive search strategy, the lack of a satisfactory approach to evaluating the RoB, and the failure to report the financial source of the included studies to prevent agenda bias. In addition, the quality of the evidence in many of the included reviews was low or very low. The results of the current study remind us of the necessity of critically assessing SR/MAs before using their findings and interpreting the results with great caution. It is recommended that journals ask the authors to complete checklists when submitting SR/MAs, and the reviewers also must consider a number of criteria in designing, conducting, and reporting their studies to move toward transparent and conclusive results.

## AUTHOR CONTRIBUTIONS

**Saeed Shahabi**: Conceptualization; data curation; formal analysis; funding acquisition; investigation; methodology; project administration; supervision; validation; visualization; writing—original draft; writing—review and editing. **Parviz Mojgani**: Conceptualization; formal analysis; methodology; supervision; validation; visualization; writing—original draft; writing—review and editing. **Kamran Bagheri Lankarani**: Conceptualization; methodology; supervision; validation; writing—original draft; writing—review and editing. **Maryam Jalali**: Conceptualization; formal analysis; investigation; methodology; project administration; supervision; visualization; writing—original draft; writing—review and editing.

## CONFLICT OF INTEREST STATEMENT

The authors declare no conflict of interest.

## TRANSPARENCY STATEMENT

The lead author Maryam Jalali affirms that this manuscript is an honest, accurate, and transparent account of the study being reported; that no important aspects of the study have been omitted; and that any discrepancies from the study as planned (and, if relevant, registered) have been explained.

## Supporting information

Supporting information.

## Data Availability

The data sets used and/or analyzed during the current study are available from the corresponding author on reasonable request.
