# Supplementary material for: The quality of systematic reviews/meta‐analyses assessing the effects of ankle–foot orthosis on clinical outcomes in stroke patients: A methodological systematic review
Source: Health Sci Rep. 2023 Mar 1;6(3):e1130. doi: 10.1002/hsr2.1130 (PMC9978320; doi:10.1002/hsr2.1130)
Supplement: Supplementary file 1 — Supporting information. [file HSR2-6-e1130-s001.docx]

**Supplementary Table 1:** Search strings

| **Search strings for PubMed** |
| --- |
| (("ankle-foot orthosis" OR "ankle foot orthosis" OR "ankle-foot orthoses" OR "ankle foot orthoses" OR (device AND orthotic) OR (devices AND orthotic) OR "Orthotic Device" OR orthoses OR orthoses OR orthosis OR (equipment AND orthopedic) OR (equipments AND orthopedic) OR "Orthopedic Equipments" OR (orthoses AND foot) OR "Foot Orthosis" OR (orthosis AND foot) OR "Foot Orthotic Devices" OR (device AND foot orthotic) OR (devices AND foot orthotic) OR "Foot Orthotic Device" OR (orthotic device AND foot) OR (orthotic devices AND foot) OR brace OR braces OR orthotic OR orthosis OR "orthopeadic support device" OR "orthotic device" OR "orthotic devices") AND (stroke OR strokes OR "Cerebrovascular Accident" OR "Cerebrovascular Accidents" OR CVA OR cvas OR "Cerebrovascular Apoplexy" OR (apoplexy AND cerebrovascular) OR (vascular accident AND brain) OR "Brain Vascular Accident" OR "Brain Vascular Accidents" OR (vascular accidents AND brain) OR "Cerebrovascular Stroke" OR "Cerebrovascular Strokes" OR (stroke AND cerebrovascular) OR (strokes AND cerebrovascular) OR apoplexy OR "Cerebral Stroke" OR "Cerebral Strokes" OR (stroke AND cerebral) OR (strokes AND cerebral) OR (stroke AND acute) OR "Acute Stroke" OR "Acute Strokes" OR (strokes AND acute) OR ("Cerebrovascular Accident" AND acute) OR "Acute Cerebrovascular Accident" OR "Acute Cerebrovascular Accidents" OR ("Cerebrovascular Accidents" AND acute) OR "acute cerebrovascular lesion" OR ("acute focal" AND "cerebral vasculopathy") OR "apoplectic stroke" OR apoplexy OR apoplexy OR "blood flow disturbance" OR "brain accident" OR "brain attack" OR "brain insult" OR "brain insultus" OR "brain ischaemic attack" OR "brain ischemic attack" OR "brain vascular accident" OR "cerebral apoplexia" OR "cerebral insult" OR "cerebral stroke" OR "cerebral vascular insufficiency" OR "cerebro vascular accident" OR "cerebrovascular arrest" OR "cerebrovascular failure" OR "cerebrovascular injury" OR "cerebrovascular insufficiency" OR "cerebrovascular insult" OR "cerebrum vascular accident" OR "cryptogenic stroke" OR "ischaemic cerebral attack" OR "ischaemic seizure" OR "ischemic cerebral attack" OR "ischemic seizure" OR "brain arterial insufficiency" OR "brain circulation disorder" OR "chronic ischaemic stroke" OR "chronic ischemic stroke" OR "ischaemia cerebri" OR "ischaemic brain disease" OR "ischaemic encephalopathy" OR "ischaemic stroke" OR "ischemia cerebri" OR "ischemic brain disease" OR "ischemic encephalopathy" OR "ischemic stroke" OR "neural ischaemia" OR "neural ischemia" OR "cerebrovascular circulation disorder") AND ("systematic review" OR review OR "meta analysis" OR meta-analysis OR "meta analyses")) |
| **Search strings for Scopus** |
| (ALL("ankle-foot orthosis") OR ALL("ankle foot orthosis") OR ALL("ankle-foot orthoses") OR ALL("ankle foot orthoses") OR (TITLE-ABS(device) AND TITLE-ABS(orthotic)) OR (TITLE-ABS(devices) AND TITLE-ABS(orthotic)) OR TITLE-ABS("Orthotic Device") OR TITLE-ABS(orthoses) OR TITLE-ABS(orthoses) OR TITLE-ABS(orthosis) OR (TITLE-ABS(equipment) AND TITLE-ABS(orthopedic)) OR (TITLE-ABS(equipments) AND TITLE-ABS(orthopedic)) OR TITLE-ABS("Orthopedic Equipments") OR (TITLE-ABS(orthoses) AND TITLE-ABS(foot)) OR TITLE-ABS("Foot Orthosis") OR (TITLE-ABS(orthosis) AND TITLE-ABS(foot)) OR TITLE-ABS("Foot Orthotic Devices") OR (TITLE-ABS(device) AND TITLE-ABS("foot orthotic")) OR (TITLE-ABS(devices) AND TITLE-ABS("foot orthotic")) OR TITLE-ABS("Foot Orthotic Device") OR (TITLE-ABS("orthotic device") AND TITLE-ABS(foot)) OR (TITLE-ABS("orthotic devices") AND TITLE-ABS(foot)) OR TITLE-ABS(brace) OR TITLE-ABS(braces) OR TITLE-ABS(orthotic) OR TITLE-ABS(orthosis) OR TITLE-ABS("orthopeadic support device") OR TITLE-ABS("orthotic device") OR TITLE-ABS("orthotic devices")) AND (TITLE-ABS(stroke) OR TITLE-ABS(strokes) OR TITLE-ABS("Cerebrovascular Accident") OR TITLE-ABS("Cerebrovascular Accidents") OR TITLE-ABS(CVA) OR TITLE-ABS(CVAS) OR TITLE-ABS("Cerebrovascular Apoplexy") OR (TITLE-ABS(apoplexy) AND TITLE-ABS(cerebrovascular)) OR (TITLE-ABS(vascular accident) AND TITLE-ABS(brain)) OR TITLE-ABS("Brain Vascular Accident") OR TITLE-ABS("Brain Vascular Accidents") OR (TITLE-ABS("vascular accidents") AND TITLE-ABS(brain)) OR TITLE-ABS("Cerebrovascular Stroke") OR TITLE-ABS("Cerebrovascular Strokes") OR (TITLE-ABS(stroke) AND TITLE-ABS(cerebrovascular)) OR (TITLE-ABS(strokes) AND TITLE-ABS(cerebrovascular)) OR TITLE-ABS(apoplexy) OR TITLE-ABS("Cerebral Stroke") OR TITLE-ABS("Cerebral Strokes") OR (TITLE-ABS(stroke) AND TITLE-ABS(cerebral)) OR (TITLE-ABS(strokes) AND TITLE-ABS(cerebral)) OR (TITLE-ABS(stroke) AND TITLE-ABS(acute)) OR TITLE-ABS("Acute Stroke") OR TITLE-ABS("Acute Strokes") OR (TITLE-ABS(strokes) AND TITLE-ABS(acute)) OR (TITLE-ABS("Cerebrovascular Accident") AND TITLE-ABS(acute)) OR TITLE-ABS("Acute Cerebrovascular Accident") OR TITLE-ABS("Acute Cerebrovascular Accidents") OR (TITLE-ABS("Cerebrovascular Accidents") AND TITLE-ABS(acute)) OR TITLE-ABS("acute cerebrovascular lesion") OR (TITLE-ABS("acute focal") AND TITLE-ABS("cerebral vasculopathy")) OR TITLE-ABS("apoplectic stroke") OR TITLE-ABS(apoplexy) OR TITLE-ABS(apoplexy) OR TITLE-ABS("blood flow disturbance") OR TITLE-ABS("brain accident") OR TITLE-ABS("brain attack") OR TITLE-ABS("brain insult") OR TITLE-ABS("brain insultus") OR TITLE-ABS("brain ischaemic attack") OR TITLE-ABS("brain ischemic attack") OR TITLE-ABS("brain vascular accident") OR TITLE-ABS("cerebral apoplexia") OR TITLE-ABS("cerebral insult") OR TITLE-ABS("cerebral stroke") OR TITLE-ABS("cerebral vascular insufficiency") OR TITLE-ABS("cerebro vascular accident") OR TITLE-ABS("cerebrovascular arrest") OR TITLE-ABS("cerebrovascular failure") OR TITLE-ABS("cerebrovascular injury") OR TITLE-ABS("cerebrovascular insufficiency") OR TITLE-ABS("cerebrovascular insult") OR TITLE-ABS("cerebrum vascular accident") OR TITLE-ABS("cryptogenic stroke") OR TITLE-ABS("ischaemic cerebral attack") OR TITLE-ABS("ischaemic seizure") OR TITLE-ABS("ischemic cerebral attack") OR TITLE-ABS("ischemic seizure") OR TITLE-ABS("brain arterial insufficiency") OR TITLE-ABS("brain circulation disorder") OR TITLE-ABS("chronic ischaemic stroke") OR TITLE-ABS("chronic ischemic stroke") OR TITLE-ABS("ischaemia cerebri") OR TITLE-ABS("ischaemic brain disease") OR TITLE-ABS("ischaemic encephalopathy") OR TITLE-ABS("ischaemic stroke") OR TITLE-ABS("ischemia cerebri") OR TITLE-ABS("ischemic brain disease") OR TITLE-ABS("ischemic encephalopathy") OR TITLE-ABS("ischemic stroke") OR TITLE-ABS("neural ischaemia") OR TITLE-ABS("neural ischemia") OR TITLE-ABS("cerebrovascular circulation disorder")) AND (TITLE-ABS("systematic review") OR TITLE-ABS(review) OR TITLE-ABS("meta analysis") OR TITLE-ABS(meta-analysis) OR TITLE-ABS("meta analyses")) |
| **Search strings for Web of Science** |
| ((TS=("ankle-foot orthosis") OR TS=("ankle foot orthosis") OR TS=("ankle-foot orthoses") OR TS=("ankle foot orthoses") OR (TS=(device) AND TS=(orthotic)) OR (TS=(devices) AND TS=(orthotic)) OR TS=("Orthotic Device") OR TS=(orthoses) OR TS=(orthoses) OR TS=(orthosis) OR (TS=(equipment) AND TS=(orthopedic)) OR (TS=(equipments) AND TS=(orthopedic)) OR TS=("Orthopedic Equipments") OR (TS=(orthoses) AND TS=(foot)) OR TS=("Foot Orthosis") OR (TS=(orthosis) AND TS=(foot)) OR TS=("Foot Orthotic Devices") OR (TS=(device) AND TS=("foot orthotic")) OR (TS=(devices) AND TS=("foot orthotic")) OR TS=("Foot Orthotic Device") OR (TS=("orthotic device") AND TS=(foot)) OR (TS=("orthotic devices") AND TS=(foot)) OR TS=(brace) OR TS=(braces) OR TS=(orthotic) OR TS=(orthosis) OR TS=("orthopeadic support device") OR TS=("orthotic device") OR TS=("orthotic devices")) AND (TS=(stroke) OR TS=(strokes) OR TS=("Cerebrovascular Accident") OR TS=("Cerebrovascular Accidents") OR TS=(CVA) OR TS=(CVAS) OR TS=("Cerebrovascular Apoplexy") OR (TS=(apoplexy) AND TS=(cerebrovascular)) OR (TS=(vascular accident) AND TS=(brain)) OR TS=("Brain Vascular Accident") OR TS=("Brain Vascular Accidents") OR (TS=("vascular accidents") AND TS=(brain)) OR TS=("Cerebrovascular Stroke") OR TS=("Cerebrovascular Strokes") OR (TS=(stroke) AND TS=(cerebrovascular)) OR (TS=(strokes) AND TS=(cerebrovascular)) OR TS=(apoplexy) OR TS=("Cerebral Stroke") OR TS=("Cerebral Strokes") OR (TS=(stroke) AND TS=(cerebral)) OR (TS=(strokes) AND TS=(cerebral)) OR (TS=(stroke) AND TS=(acute)) OR TS=("Acute Stroke") OR TS=("Acute Strokes") OR (TS=(strokes) AND TS=(acute)) OR (TS=("Cerebrovascular Accident") AND TS=(acute)) OR TS=("Acute Cerebrovascular Accident") OR TS=("Acute Cerebrovascular Accidents") OR (TS=("Cerebrovascular Accidents") AND TS=(acute)) OR TS=("acute cerebrovascular lesion") OR (TS=("acute focal") AND TS=("cerebral vasculopathy")) OR TS=("apoplectic stroke") OR TS=(apoplexy) OR TS=(apoplexy) OR TS=("blood flow disturbance") OR TS=("brain accident") OR TS=("brain attack") OR TS=("brain insult") OR TS=("brain insultus") OR TS=("brain ischaemic attack") OR TS=("brain ischemic attack") OR TS=("brain vascular accident") OR TS=("cerebral apoplexia") OR TS=("cerebral insult") OR TS=("cerebral stroke") OR TS=("cerebral vascular insufficiency") OR TS=("cerebro vascular accident") OR TS=("cerebrovascular arrest") OR TS=("cerebrovascular failure") OR TS= ("cerebrovascular injury") OR TS=("cerebrovascular insufficiency") OR TS=("cerebrovascular insult") OR TS=("cerebrum vascular accident") OR TS=("cryptogenic stroke") OR TS=("ischaemic cerebral attack") OR TS=("ischaemic seizure") OR TS=("ischemic cerebral attack") OR TS=("ischemic seizure") OR TS=("brain arterial insufficiency") OR TS=("brain circulation disorder") OR TS=("chronic ischaemic stroke") OR TS=("chronic ischemic stroke") OR TS=("ischaemia cerebri") OR TS=("ischaemic brain disease") OR TS=("ischaemic encephalopathy") OR TS=("ischaemic stroke") OR TS=("ischemia cerebri") OR TS=("ischemic brain disease") OR TS=("ischemic encephalopathy") OR TS=("ischemic stroke") OR TS=("neural ischaemia") OR TS=("neural ischemia") OR TS=("cerebrovascular circulation disorder")) AND (TS=("systematic review") OR TS=(review) OR TS=("meta analysis") OR TS=(meta-analysis) OR TS=("meta analyses")) |
| **Search strings for Embase** |
| ('ankle-foot orthosis':ti,ab OR 'ankle foot orthosis':ti,ab OR 'ankle-foot orthoses':ti,ab OR 'ankle foot orthoses':ti,ab OR (device:ti,ab AND orthotic:ti,ab) OR (devices:ti,ab AND orthotic:ti,ab) OR orthoses:ti,ab OR orthosis:ti,ab OR (equipment:ti,ab AND orthopedic:ti,ab) OR (equipments:ti,ab AND orthopedic:ti,ab) OR 'orthopedic equipments':ti,ab OR (orthoses:ti,ab AND foot:ti,ab) OR 'foot orthosis':ti,ab OR (orthosis:ti,ab AND foot:ti,ab) OR 'foot orthotic devices':ti,ab OR (device:ti,ab AND 'foot orthotic':ti,ab) OR (devices:ti,ab AND 'foot orthotic':ti,ab) OR 'foot orthotic device':ti,ab OR ('orthotic device':ti,ab AND foot:ti,ab) OR ('orthotic devices':ti,ab AND foot:ti,ab) OR brace:ti,ab OR braces:ti,ab OR orthotic:ti,ab OR 'orthopeadic support device':ti,ab OR 'orthotic device':ti,ab OR 'orthotic devices':ti,ab) AND (stroke:ti,ab OR strokes:ti,ab OR 'cerebrovascular accident':ti,ab OR 'cerebrovascular accidents':ti,ab OR cva:ti,ab OR cvas:ti,ab OR 'cerebrovascular apoplexy':ti,ab OR (apoplexy:ti,ab AND cerebrovascular:ti,ab) OR ('vascular accident':ti,ab AND brain:ti,ab) OR 'brain vascular accidents':ti,ab OR ('vascular accidents':ti,ab AND brain:ti,ab) OR 'cerebrovascular stroke':ti,ab OR 'cerebrovascular strokes':ti,ab OR (stroke:ti,ab AND cerebrovascular:ti,ab) OR (strokes:ti,ab AND cerebrovascular:ti,ab) OR 'cerebral strokes':ti,ab OR (stroke:ti,ab AND cerebral:ti,ab) OR (strokes:ti,ab AND cerebral:ti,ab) OR (stroke:ti,ab AND acute:ti,ab) OR 'acute stroke':ti,ab OR 'acute strokes':ti,ab OR (strokes:ti,ab AND acute:ti,ab) OR ('cerebrovascular accident':ti,ab AND acute:ti,ab) OR 'acute cerebrovascular accident':ti,ab OR 'acute cerebrovascular accidents':ti,ab OR ('cerebrovascular accidents':ti,ab AND acute:ti,ab) OR 'acute cerebrovascular lesion':ti,ab OR ('acute focal':ti,ab AND 'cerebral vasculopathy':ti,ab) OR 'apoplectic stroke':ti,ab OR apoplexy:ti,ab OR 'blood flow disturbance':ti,ab OR 'brain accident':ti,ab OR 'brain attack':ti,ab OR 'brain insult':ti,ab OR 'brain insultus':ti,ab OR 'brain ischaemic attack':ti,ab OR 'brain ischemic attack':ti,ab OR 'brain vascular accident':ti,ab OR 'cerebral apoplexia':ti,ab OR 'cerebral insult':ti,ab OR 'cerebral stroke':ti,ab OR 'cerebral vascular insufficiency':ti,ab OR 'cerebro vascular accident':ti,ab OR 'cerebrovascular arrest':ti,ab OR 'cerebrovascular failure':ti,ab OR 'cerebrovascular injury':ti,ab OR 'cerebrovascular insufficiency':ti,ab OR 'cerebrovascular insult':ti,ab OR 'cerebrum vascular accident':ti,ab OR 'cryptogenic stroke':ti,ab OR 'ischaemic cerebral attack':ti,ab OR 'ischaemic seizure':ti,ab OR 'ischemic cerebral attack':ti,ab OR 'ischemic seizure':ti,ab OR 'brain arterial insufficiency':ti,ab OR 'brain circulation disorder':ti,ab OR 'chronic ischaemic stroke':ti,ab OR 'chronic ischemic stroke':ti,ab OR 'ischaemia cerebri':ti,ab OR 'ischaemic brain disease':ti,ab OR 'ischaemic encephalopathy':ti,ab OR 'ischaemic stroke':ti,ab OR 'ischemia cerebri':ti,ab OR 'ischemic brain disease':ti,ab OR 'ischemic encephalopathy':ti,ab OR 'ischemic stroke':ti,ab OR 'neural ischaemia':ti,ab OR 'neural ischemia':ti,ab OR 'cerebrovascular circulation disorder':ti,ab) AND ('systematic review':ti,ab OR review:ti,ab OR 'meta analysis':ti,ab OR meta-analysis:ti,ab OR 'meta analyses':ti,ab)) |

**Supplementary Figure 1.** Number of review studies in different years

**
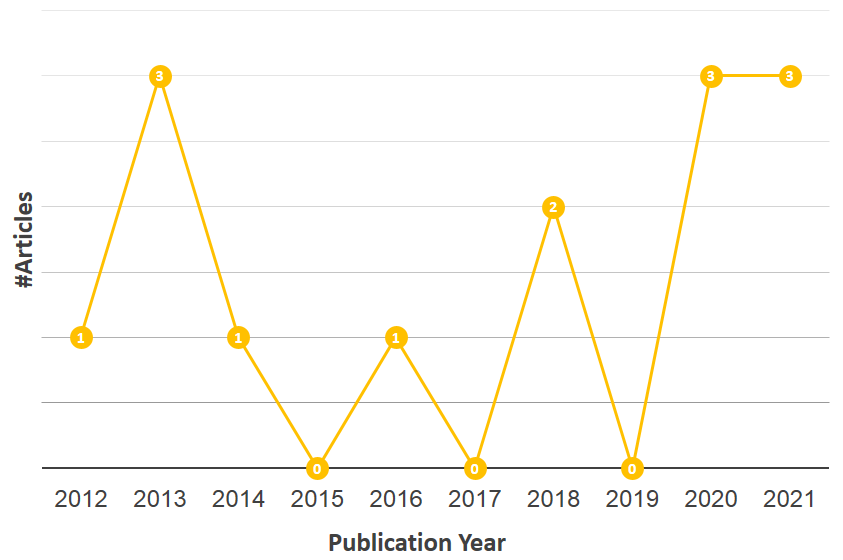
**

**Supplementary Table 2.** Excluded studies with exclusion reasons

| Author (year) | Title | Reason for exclusion |
| --- | --- | --- |
| Mahmoudi et al. (2021) | The Effects of Electrical Stimulation of Lower Extremity Muscles on Balance in Stroke Patients: A Systematic Review of Literatures | Other interventions |
| Ramstrand & Stevens  (2021) | Clinical outcome measures to evaluate the effects of orthotic management post-stroke: a systematic review | Other interventions |
| Figueiredo et al.  (2019) | Outcome measures and motion capture systems for assessing lower limb orthosis-based interventions after stroke: a systematic review. | Other interventions |
| Lefeber et al. (2019) | Energy consumption and cost during walking with different modalities of assistance after stroke: a systematic review and meta-analysis | Other interventions |
| Totah et al.  (2019) | The Impact of Ankle-Foot Orthosis Stiffness on Gait: A Systematic Literature Review | Other population |
| Dee et al.  (2018) | A systematic review of physical rehabilitation interventions for stroke in low and lower-middle income countries | Other interventions |
| Healy et al.  (2018) | A systematic review of randomised controlled trials assessing effectiveness of prosthetic and orthotic interventions | Other interventions and population |
| Wang et al.  (2019) | Use of Kinesio taping in lower-extremity rehabilitation of post-stroke patients: A systematic review and meta-analysis | Other interventions |
| Mahmood et al.  (2019) | Effect of Transcutaneous Electrical Nerve Stimulation on Spasticity in Adults With Stroke: A Systematic Review and Meta-analysis | Other interventions |
| Hu et al.  (2019) | Kinesio Taping for Balance Function after Stroke: A Systematic Review and Meta-Analysis | Other interventions |
| Arienti et al.  (2019) | Rehabilitation interventions for improving balance following stroke: An overview of systematic reviews | Other interventions/Overview study |
| Hong et al.  (2018) | Effectiveness of Neuromuscular Electrical Stimulation on Lower Limbs of Patients With Hemiplegia After Chronic Stroke: A Systematic Review | Other interventions |
| Takeda et al.  (2017) | Review of devices used in neuromuscular electrical stimulation for stroke rehabilitation | Other interventions/No systematic review |
| Wu et al.  (2016) | Effectiveness of Botulinum Toxin for Lower Limbs Spasticity after Stroke: A Systematic Review and Meta-Analysis | Other interventions |
| Grampurohit et al.  (2015) | Efficacy of adhesive taping as an adjunt to physical rehabilitation to influence outcomes post-stroke: a systematic review | Other interventions |
| Dunn et al.  (2015) | Protocol Variations and Six-Minute Walk Test Performance in Stroke Survivors: A Systematic Review with Meta-Analysis | Other interventions |
| Dunning et al. (2015) | Peroneal stimulation for foot drop after stroke: a systematic review | Other interventions |
| Shrivastava et al.  (2014) | Ankle foot orthosis for walking in stroke rehabilitation | Protocol study |
| Phadke et al.  (2014) | The impact of post-stroke spasticity and botulinum toxin on standing balance: a systematic review | Other interventions |
| Esquenazi et al.  (2009) | The Effect of an Ankle-Foot Orthosis on Temporal Spatial Parameters and Asymmetry of Gait in Hemiparetic Patients | No systematic review |
| Peppen et al.  (2004) | The impact of physical therapy on functional outcomes after stroke: what's the evidence? | Other interventions |
| Kottink et al.  (2003) | The Orthotic Effect of Functional Electrical Stimulation on the Improvement of Walking in Stroke Patients with a Dropped Foot: A Systematic Review | Other interventions |
| Fish et al.  (2001) | Lower Extremity Orthoses And Applications For Rehabilitation Populations | No systematic review |

**Supplementary Table 3.** ROBIS results

| **References** | **Domain 1: Study Eligibility Criteria** | **Domain 2: Identification and Selection of Studies** | **Domain 3: Data Collection and Study Appraisal** | **Domain 4: Synthesis and Findings** | **Risk of Bias in the Review** |
| --- | --- | --- | --- | --- | --- |
| Choo & Chang  (2021) | Unclear | Low | High | High | Unclear |
| Daryabor et al.  (2021) | Low | Unclear | High | Unclear | Unclear |
| Daryabor et al.  (2020) | High | High | High | Unclear | High |
| Daryabor et al.  (2018) | High | High | Low | Unclear | Unclear |
| Ferreira et al.  (2013) | High | High | High | High | High |
| Hollands et al.  (2012) | High | Low | High | Unclear | Unclear |
| Nascimento et al.  (2020) | Low | High | Low | Unclear | Low |
| Padilla et al.  (2014) | High | Low | High | Unclear | Low |
| Prenton et al.  (2018) | Low | Low | Unclear | High | Unclear |
| Prenton et al.  (2016) | Low | Low | Unclear | High | Unclear |
| Shahabi et al.  (2020) | Low | Low | Unclear | Unclear | Low |
| Tyson et al.  (2013a) | High | Low | Low | Unclear | Unclear |
| Tyson et al.  (2013b) | High | Unclear | Unclear | Unclear | Unclear |
| Wada et al.  (2021) | Low | Low | Unclear | High | Unclear |
